# *APOL1* genotypes: Do they contribute to ethnicity-associated biological health inequalities in pregnancy?

**DOI:** 10.1177/1753495X211043750

**Published:** 2021-10-20

**Authors:** Priscilla Smith, Kate Bramham

**Affiliations:** King’s Kidney Care, 8948 King’s College Hospital NHS Foundation Trust, London, UK Email: Priscilla.smith@kcl.ac.uk; Department of Women and Children’s Health, King’s College London, London, UK

**Keywords:** Apolipoprotein 1, ethnicity, pregnancy, preeclampsia, chronic kidney disease

## Abstract

Inferior health outcomes for people of African and Afro-Caribbean ancestry compared to those of European ancestry are well recognised. There is a disproportionate impact within these communities compared to other ethnic groups including pregnancy outcomes, hypertension, kidney disease and diabetes. The ‘Black Lives Matter’ movement has highlighted that it is imperative to examine all factors contributing to this inequity and to strive to explore multifaceted ways, including social, economic, psychological and biological to improve overall health equity. It is within this context that we discuss the novel finding of Apolipoprotein 1 genetic polymorphisms which have been identified in some populations of African ancestry. We will explore the history and evolutionary advantages of Apolipoprotein 1 polymorphisms and the pathophysiology resulting from these adaptations and examine the impact of Apolipoprotein 1 on pregnancy outcomes, the risks and benefits of screening for high-risk Apolipoprotein 1 alleles in black communities and potential treatments currently being investigated.

Of all the forms of inequality, injustice in health is the most shocking and the most inhuman.

Martin Luther King, 1966

## Introduction

Inferior health outcomes for people of African and Afro-Caribbean ancestry compared to those of European ancestry are well recognised. There is a disproportionate impact within these communities compared to other ethnic groups with access to the same health systems and standards of care for diverse conditions including pregnancy outcomes,^1^ hypertension, kidney disease or diabetes.^2–4^ The ‘Black Lives Matter’^5^ movement has highlighted that it is imperative to examine all factors contributing to this inequity and to strive to explore multifaceted ways, including social, economic, psychological and biological to improve overall health equity. It is within this context that we discuss the novel finding of Apolipoprotein 1 (*APOL1*) genetic polymorphisms which have been identified in some populations of African ancestry.^6^ We will explore the history and evolutionary advantages of *APOL1* polymorphisms and the pathophysiology resulting from these adaptations and examine the impact of *APOL1* on pregnancy outcomes, the risks and benefits of screening for high-risk *APOL1* alleles in black communities and potential treatments currently being investigated.

## History

*APOL1* gene polymorphisms, which are present in people of African ancestry, were only identified and studied during the last 10 years.^7,8^ The APOL gene cluster (*APOL1-6*) is found on chromosome 22^9^ and is present solely in humans and few higher primates.^10^ *APOL1* is the only APOL gene with signal peptide enabling both circulating and intracellular effects,^8^ and is expressed in a number of cell types including liver (the source of secreted circulating apolipoprotein-L1), placental and renal parenchymal cells^11^ but the absence of expression is not associated with a disease phenotype.^12^

**Figure 1.**
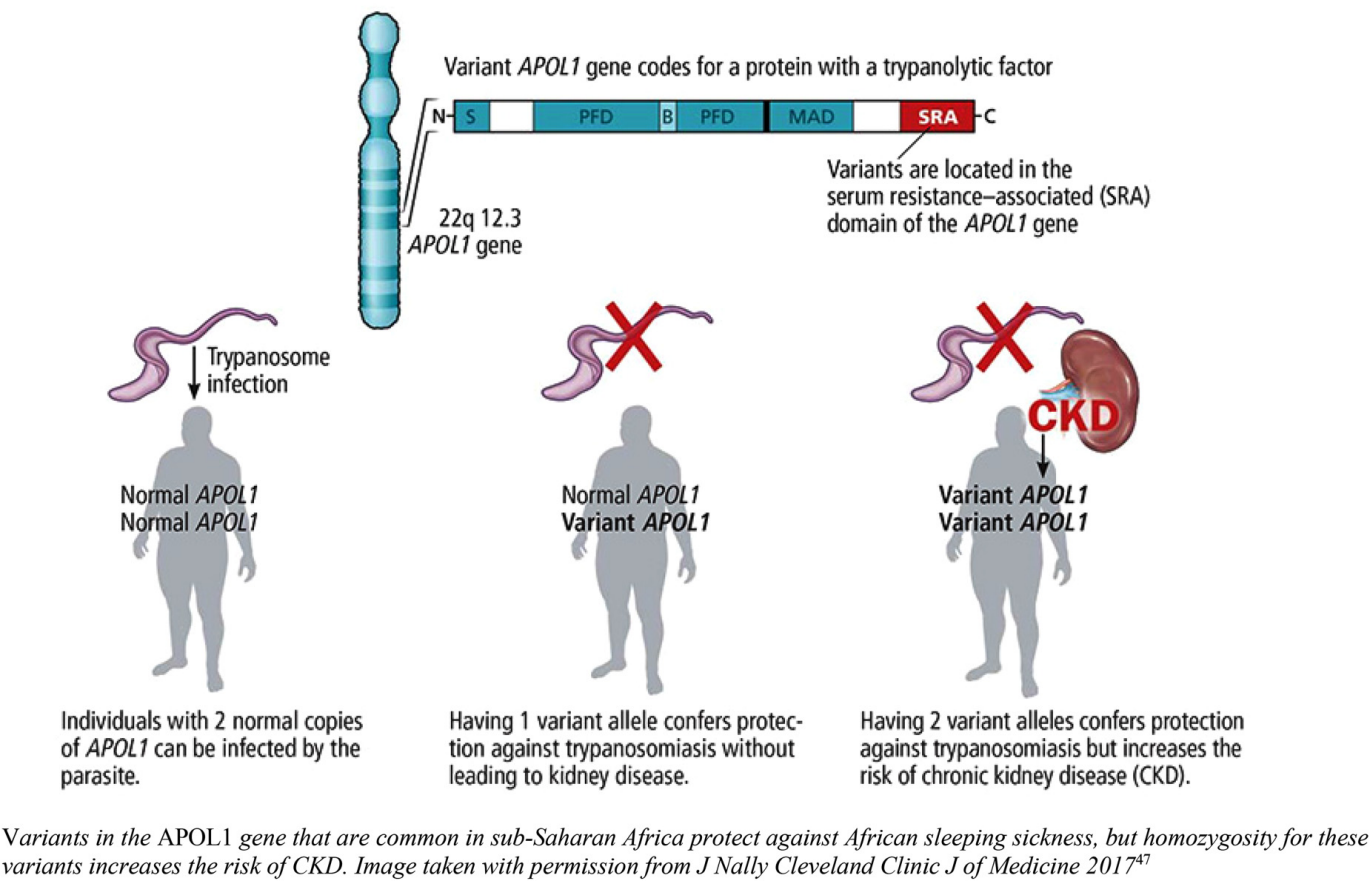
*APOL1* variants and effects. Variants in the APOL1 gene that are common in sub-Saharan Africa protect against African sleeping sickness, but homozygosity for these variants increases the risk of CKD. The image was taken with permission from J Nally Cleveland Clinic J of Medicine 2017.^13^

APOL1: apolipoprotein 1; CKD: chronic kidney disease.

The evolutionary advantage of circulating *APOL1* protein was due to protection against *Trypanosoma brucei* species (including one which causes African sleeping sickness) through trypanolytic effect. Counter evolution of serum resistance-associated protein by *Trypanosoma brucei rhodesiense* and *T. b. gambiense*-specific glycoprotein (TgsGP) by *T. b. gambiense* which both block the action of *APOL1* is thought to have led to the development of *APOL1* variants G1 (two missense variants: rs73885319 and rs60910145) and G2 (6 bp deletion, rs71785313) to prevent parasitic evasion of apolipoprotein-L1^11^ (see Figure 1).

Homozygotes for G1 and G2 risk alleles or compound heterozygotes have an increased risk of kidney disease and progression to end-stage kidney disease (ESKD) (see Table 1).^6^ These risk alleles are present in those with ‘recent’ African ancestry (last 10–50,000 years) and are most prevalent in West Africa. Due to historical slave trading, there is also a high prevalence of *APOL1* risk alleles in African-American populations.

**Table 1.** *APOL1* and CKD association studies.

Study citation	Cohort size	Outcomes	Stat	*P*-value
Genovese et al.**^7^**	2055	End stage kidney disease^e^	OR 7.3 (5.6–9.5)	
	385	FSGS^f^	OR 10.5 (6.0–18.4)	
Kopp et al.^21^	1378	HIVAN^a^	OR 29.2 (13.1–68.5)	6 × 10^−22^
		FSGS^a^	OR 16.9 (11–26.5)	1.3 × 10^−48^
Peralta et al.^25^	3030	Albuminuria development^b^	OR 3.50 (2.14–5.71)	<0.05
		GFR decline per year^b^	0.45% ml/min/1.73 m^2^ (0.21–0.68)	<0.05
Parsa (AASK) 2013^26^	693	End stage kidney disease	HR 2.21 (1.56–3.14)	<0.001
		Composite renal outcome (ESKD + doubling S creat)	HR 2.03 (1.5–2.74)	<0.001
Parsa (CRIC) 2013^26^	2955	Composite renal outcome^c^	HR 2.84 (1.84–4.38)	<0.001
		Composite renal outcome^d^	HR 1.95 (1.39–2.73)	<0.001
		GFR decline per year^c^	−0.81 (−1.26 to −0.35)	<0.001
		GFR decline per year^d^	−0.79 (−1.41 to −0.17)	<0.01

AASK: African-American Study of Kidney Disease and Hypertension; APOL1: apolipoprotein 1; CKD: chronic kidney disease; CRIC: Chronic Renal Insufficiency Cohort; ESKD: End-stage kidney disease; FSGS: Focal segmental glomerulosclerosis; GFR: Glomerular Filtration Rate; HR: Hazard Ratio; HIVAN: HIV-associated nephropathy; OR: Odds Ratio.

^a^ 2 APOL1 risk alleles vs. 1/0.

^b^ 2 APOL1 risk alleles vs. White.

^c^ 2 APOL1 risk alleles vs. White – non-diabetics.

^d^ High risk APOL1 vs. Whites – diabetics.

^e^ ESKD in hypertensives with 2 risk alleles vs. 0 risk alleles.

^f^ 2 risk alleles vs. 0/1 risk alleles.

## Pathophysiology

*APOL1* protein in its circulating form does not appear to impact on development of kidney disease.^14^ Studies of kidney transplant patients have demonstrated that recipients with *APOL1* risk alleles do not develop recurrent disease or show reduced graft survival when transplanted with *APOL1* G0/G0 kidneys.^15^ Conversely, *APOL1* G1/G2 donor kidneys transplanted into low-risk recipients have higher rates of graft failure and reduced graft survival time.^16–18^ Whilst exact pathophysiological mechanisms are still being elucidated, the expression of *APOL1* mRNA in renal parenchymal cells, in particular podocytes, is associated with cell damage from membrane pore formation.^19^ This results in reduced podocyte density possibly also due to increased senescence and apoptosis mediated by *APOL1* expression. However, this process does not invariably lead to kidney disease^10^ and it is hypothesised that rather than true Mendelian inheritance these polymorphisms cause disease via a ‘second hit’ mechanism.^20^

Viral infections (especially HIV), drugs (interferon), low nephron mass (e.g. from prematurity), diabetes and obesity are all potential ‘second hits’ to the kidney which may contribute to the development of clinical disease. Most *APOL1* related kidney disease presents with albuminuria with histological changes consistent with focal segmental glomerulosclerosis (FSGS) on biopsy,^21^ but *APOL1* risk alleles are also considered to underlie ‘hypertensive nephrosclerosis’ in people of African or Afro-Caribbean ancestry. Many of these patients may not receive a kidney biopsy but are known to progress more rapidly to ESKD even in the presence of tightly controlled blood pressure.^22–24^

## Clinical effects

The presence of two *APOL1* risk alleles has been strongly associated with the development of chronic kidney disease (CKD) in several large cohort studies.^6,19,25,26^ The odds ratios for development of FSGS and HIV-associated nephropathy (HIVAN) in those with risk alleles are 17 and 29, respectively, compared to those without risk alleles (see Table 1).^19^ Studies from the US have reported that over 70% of African Americans with FSGS and HIVAN have two *APOL1* risk alleles compared to 12% healthy controls.^10,19,27^

Conversely, there appears to be a survival advantage of *APOL1* risk alleles, after kidney function has reached end stage. Reduction in all cause and cardiovascular deaths are reported in dialysis patients of African ancestry with ESKD attributed to diabetes and/or *APOL1* risk alleles compared to dialysis patients of white ethnicity,^28^ but ethnicity differences in outcomes were not significant between patients with ESKD attributed to non-*APOL1* mediated disease (i.e. other immune glomerulonephritis). Previous cohort studies have suggested an increased cardiovascular risk with risk *APOL1* alleles,^29^ but the US Million Veteran study^30^ has demonstrated that augmented cardiovascular risk is likely minimal and mediated through vascular pathology associated with kidney disease rather than *APOL1* genotype being an independent cardiovascular risk factor.

## *APOL1* and pregnancy

Pre-eclampsia risk, with associated maternal and fetal morbidity and mortality, is increased in women of African ancestry,^31^ and *APOL1* risk genotypes during pregnancy^32^ for some women may be contributory through diverse mechanisms.

A novel transgenic mouse *APOL1* G2 model study reported increased rates of pre-eclampsia and decreased podocyte density with aging, despite not developing overt kidney disease.^33^ *APOL1* mRNA is strongly expressed in placental tissue^34^ and pregnancies with G2 homozygous mouse offspring had higher rates of pregnancy complications, suggesting a role of an infant rather than maternal genotype. These findings are aligned with previous clinical observations of paternal association with development of pre-eclampsia including in those of African ancestry.^31,35,36^

**Figure 2.**
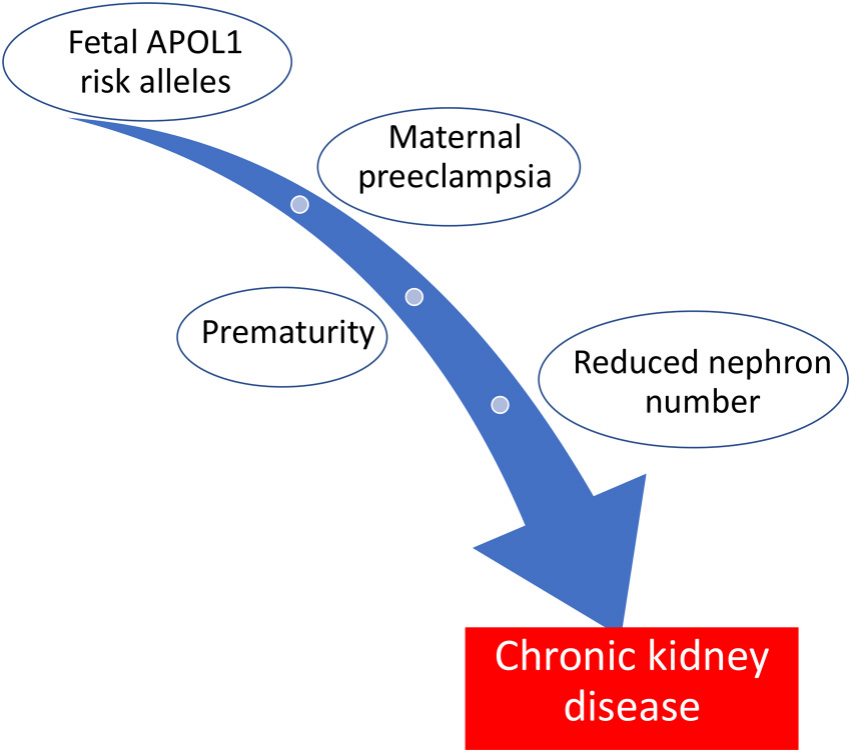
‘Two Hit’ hypothesis of early CKD in offspring with *APOL1* risk alleles.

APOL1: apolipoprotein 1; CKD: chronic kidney disease.

The relationship between *APOL1* risk alleles and adverse pregnancy outcomes (see Table 2) has been further supported by observational cohorts from South Africa, in which early-onset pre-eclampsia was significantly associated with maternal *APOL1* G1 genotype in a dominant model.^37^ Two large USA cohorts^38^ of black women with pre-eclampsia could not demonstrate an association with maternal *APOL1* genotype due to low case numbers and non-significant results. This may reflect a phenotypic difference between these black populations which has not been fully explored. The prevalence of high-risk fetal *APOL1* genotypes in the USA cohorts was two-fold higher in pregnancies complicated by pre-eclampsia compared to ethnically matched controls who did not develop pre-eclampsia. Lower rates of maternal high-risk *APOL1* genotypes in women with pre-eclampsia compared to rates in their fetuses, although not reaching statistical significance, may suggest a further impact of discordant maternal/fetal genotypes. Prematurity secondary to pre-eclampsia in infants carrying high-risk genotypes could be a ‘second hit’ leading to a higher incidence of early onset, possibly more severe, kidney disease in the offspring (Figure 2).^39,40^ Identification of these at-risk infants may provide a target for early screening and interventions to prevent or delay progression to ESKD.

**Table 2.** *APOL1* and pregnancy studies.

Study citation	Cohort size	Outcomes	Odds ratio (95% CI)	*P*-value
Bruggeman et al.^33^ JASN	N/A	Reduced nephrons and pre-eclampsia development in G2 transgenic mice.	–	–
Reidy et al.^38^**^a^**	121	Fetal high risk *APOL1* genotype	1.84 (1.11–2.93)	<0.05
		Maternal high risk *APOL1* genotype	0.72 (0.11–2.49)	–
Reidy et al.^38^**^b^**	921	Fetal high risk *APOL1* genotype	1.92 (1.03–3.43)	<0.05
		Maternal high risk *APOL1* genotype	0.54 (0.21–1.17)	–
Miller et al.^32^**^c^**	677	Pre-eclampsia association with fetal genotype^e^	1.41 (1.04–1.93)	0.029
Thakoordeen-Reddy et al.^37^**^d^**	428	Early-onset pre-eclampsia association with maternal G1 genotype^e^	1.88 (1.02–3.45)	<0.04

^a^ Pre-eclampsia case only, New York.

^b^ Pre-eclampsia case-controlled, Tennessee.

^c^ Ohio.

^d^ South Africa.

^e^ Dominant inheritance model.

CKD is one of the most prevalent conditions in women of child-bearing age and is increasing globally.^41^ As women more frequently delay pregnancy until older age, there is a further increase in prevalent CKD as well as risk factors for its development, such as diabetes, obesity and hypertension. Given the asymptomatic nature of CKD in early stages, routine screening in pregnancy can identify diseases which may not otherwise have been recognised including *APOL1* related kidney disease. Early CKD is an independent risk factor for adverse pregnancy outcomes.^42,43^ *APOL1* renal disease is frequently associated with chronic hypertension which is itself a risk factor for poor pregnancy outcomes.^44^ Furthermore, renal hyperfiltration which occurs as a physiological adaptation in pregnancy may further stress kidneys with previously unrecognised disease leading to a pathological increase in proteinuria and acceleration of the renal functional decline.

## Treatment options

There is currently no targeted therapy for *APOL1* mediated kidney disease, but several are in development. Standard management includes salt restriction, fluid management with diuretics and renin-angiotensin-aldosterone system blockade. Response to steroids and other immunosuppression varies.^45^ Early diagnosis and initiation of highly active antiretroviral therapy for HIV may reduce or delay development of HIVAN in susceptible individuals.

## Screening

Screening for any genetically mediated health condition is costly, has several inherent risks and is only justified when the early diagnosis can improve outcomes. In the absence of a direct therapy for *APOL1* mediated disease, screening for high-risk genotypes has not been explored. Although early identification of CKD through ongoing surveillance of those at risk could slow progression to ESKD and improve symptom management, screening is likely to be costly. In addition, there are concerns about impacts on health and life insurance as well as a perceived sense of own health, particularly for those who do not have a ‘second hit’ and develop disease. However, distribution of knowledge is important and even if widespread screening is not offered potentially affected communities are keen to raise awareness of the potential role of *APOL1* risk alleles in the development of kidney disease.^46^

## Future research

The development of targeted therapy for *APOL1* mediated renal disease is a priority that is being investigated in a current commercial Phase 2 trial by Vertex Pharmaceuticals^47^ of a new medication to block *APOL1* mediated podocyte damage in patients with primary FSGS, nephrotic level proteinuria and two *APOL1* risk alleles. Other investigators are also exploring potential treatments for FSGS, but without targeting the *APOL1* pathway.

For maternity research, there remain opportunities for a greater understanding of the mechanisms and associated risks in pregnancy from *APOL1* polymorphisms. Screening for risk alleles in women of African ancestry could enable better risk stratification and the role of aspirin prophylaxis could be explored. Furthermore, knowledge of *APOL1* risk alleles in their offspring, particularly in those born pre-term could enable targeted preservation of reduced nephron numbers.

While the last decade has seen a rapidly advancing understanding of *APOL1* there remain many unanswered questions and more studies are needed to determine the role of *APOL1* risk alleles in the pathophysiology, risk stratification and or/therapeutic target for both kidney disease and management of pre-eclampsia.
